# Novel Insights reveal Anti-microbial Gene Regulation of Piglet Intestine Immune in response to *Clostridium perfringens* Infection

**DOI:** 10.1038/s41598-018-37898-5

**Published:** 2019-02-13

**Authors:** Xiao Yu Huang, Wen Yang Sun, Zun Qiang Yan, Hai Ren Shi, Qiao Li Yang, Peng Fei Wang, Sheng Gui Li, Li Xia Liu, Sheng Guo Zhao, Shuang Bao Gun

**Affiliations:** 1 College of Animal Science and Technology, Gansu Agricultural University, Lanzhou, 730070 China; 2 College of Life Science and Engineering, Northwest Minzu University, Lanzhou, 730030 China; 3 Gansu Research Center for Swine Production Engineering and Technology, Lanzhou, 730070 China

## Abstract

LncRNA play important roles in regulation of host immune and inflammation responses in defending bacterial infection. *Clostridium perfringens* (*C*. *perfringens*) type C is one of primary bacteria leading to piglet diarrhea and other intestinal inflammatory diseases. For the differences of host immune capacity, individuals usually show resistance and susceptibility to bacterial infection. However, whether and how lncRNAs involved in modulating host immune resistance have not been reported. We have investigated the expression patterns of ileum lncRNAs of 7-day-old piglets infected by *C*. *perfringens* type C through RNA sequencing. A total of 16 lncRNAs and 126 mRNAs were significantly differentially expressed in resistance (IR) and susceptibility (IS) groups. Many lncRNAs and mRNAs were identified to regulate resistance and susceptibility of piglets through immune related pathways. Five lncRNAs may have potential function on regulating the expressions of cytokines, these lncRNAs and cytokines work together to co-regulated piglet immune response to *C*. *perfringens*, affecting host resistance and susceptibility. These results provide valuable information for understanding the functions of lncRNA and mRNA in affecting piglet diarrhea resistance of defensing to *C*. *perfringens* type C, these lncRNAs and mRNAs may be used as the important biomarkers for decreasing *C*. *perfringens* spread and diseases in human and piglets.

## Introduction

Diarrhea is one of the important reasons leading piglet death, causing the huge economic losses in worldwide pig industries, especially the newborn and suckling piglets^1^. Recently, *Clostridium perfringens* (*C*. *perfringens*) type C is considered as an increasingly pathogenic bacteria of pig source and one of the important causes for high morbidity and mortality in neonatal piglet diarrhea^2–4^, and have become the substantial problem to hinder the health development of livestock industry. *C*. *perfringens* type C includes two types of toxins, α and β, which have been proposed to act as spreading factors that destroy the physical properties of tissue matrices and intercellular spaces, thereby aiding in the spread of bacteria within host^5^.

*C*. *perfringens* type C is also a common part of microbiota in pig intestinal tract, sows can transfer this bacterium to piglets by breast milk and feces. Clinical diseases caused by *C*. *perfringens* type C can be divided into acute and chronic course. The acute course mainly causes sudden death of 7-day-old neonatal piglets with characteristics of depression and bloody diarrhea. While chronic disease can persist more than one week, which is characterized by intermittent or persistent diarrhea with or without blood and dehydration. Generally, it affects the growth and development of sick piglets, even stiff pigs and death^3^. The contaminated pig is considered as the dangerous infector of *C*. *perfringens* type C, becoming an important cause of foodborne illness and zoonotic disease^6^. Human can be usually acquired through the consumption of *C*. *perfringens-*contaminated pork products or the direct contact with infected piglets^7^. Decreasing the prevalence and severity of *C*. *perfringens* in pig herds may effectively reduce spread and control transmission of bacteria from pig products to humans and to the environment, especially in large pork-producing and consuming countries.

In the process of bacterial infections, the severity of infection is impacted by pathogenicity of microbiota and its interaction with host immunity defense system^8^. The hypoimmune piglets are weaker and more susceptible to *C*. *perfringens* type C infection than hyperimmune ones for the lower immunity defense system. Currently, few frontier views are available to treat or reverse *C*. *perfringens* type C infection disease, the main reason is the poor understanding on the regulatory mechanism of host defense against *C*. *perfringens* type C infection. Therefore, exploring the potential mechanism of different resistances in piglets infected by *C*. *perfringens* type C will help to screen highly resistant piglets to decrease death rate, which may provide the new slight in methods of preventing and treating these infectious diseases.

LncRNAs are a type of transcripts with a length of more than 200 nucleotides and with no obvious potential to encode functional protein. Specific lncRNAs have been clearly known to participate in some important biological processes, such as development^9,10^, posttranscriptional regulation^11^, and immune diseases^12,13^, which have been drawn the increasing attention. Recently, identifying functional mRNAs of hosts regulated *C*. *perfringens* infection are clearly under the way, such as broiler chicken^14,15^. As the important posttranscriptional pathogenesis, these transcripts and their associated orchestrated networks are implicated in mediating complex pathological mechanisms of *C*. *perfringens* infection. To date, little studies have examined the dysregulated lncRNAs and their target genes in regulating resistance and susceptibility of piglets exposed to *C*. *perfringens* type C.

This study aims to identify and compare ileum lncRNA and mRNA expression profiles of resistant and sensitive piglets infected by *C*. *perfringens* type C infection using Ribo-Zero RNA-seq. An amount of differentially expressed lncRNAs and mRNAs were identified between resistant and sensitive piglets, which might play important roles in regulating piglet resistance to *C*. *perfringens* type C infection, though the potential roles of lncRNAs should be further validated. These results propose as a reliable model for exploring host resistance mechanisms in defensing *C*. *perfringens* type C infection, which may provide valuable foundation for further breeding diarrhea-resistance piglet strain.

## Materials and Methods

### Bacterial culture

The *C*. *perfringens* type C strain (CVCC 2032) was obtained from the China Veterinary Culture Collection Center and used in this study. The bacterium was cultured at 37 °C in the bouillon culture-medium (HopeBio, Qingdao, China) for 16 h with shaking before used for infection. The colony-forming units (CFUs) of *C*. *perfringens* type C was determined by plate colony counting method, and finally an expected concentration of 1 × 10^9^ CFU/mL *C*. *perfringens* type C medium was used to inoculate piglets.

### Animal experiment

All procedures described here were approved by the experimental license from Gansu Research Center of Swine Production Engineering and Technology, Gansu Agricultural University, in agreement with the relevant guidelines and regulations imposed by the Administration of Affairs Concerning Experimental Animals. Animals were humanely sacrificed as necessary to ameliorate suffering.

The candidate piglets were the descendants of seronegative Yorkshire sows × Landrace boar from health nucleus herd (confirmed by history and seronegative sows) in Dingxi city, Gansu province of China. Fecal samples of all piglets were collected and detected negative for *Escherichia coli*, *Salmonella* and *C*. *perfringens* tested by commercial enzyme-linked immuno sorbent assay (ELISA) kits (Jiancheng Bioengineering Institute, Nanjing, China) at the times of selection, transportation and inoculation. Finally, a total of 30 suckling piglets at 7 days old were screened, 5 of 30 piglets were randomly selected as control group (IC), the remaining 25 piglets were challenged by oral gavage of 1 mL 1 × 10^9^ CFU/mL *C*. *perfringens* type C medium for five consecutive days. Every piglet was housed in one pen separately to avoid cross infection, and they were raised in appropriate condition of climate-controlled and fully isolation, receiving water and diets *ad libitum*. During the period of infection, piglets were monitored for clinical signs and fecal consistency 3–4 times daily. Fecal consistency was scored based on the visual observation of symptoms traits: 0 = normal, solid feces, 1 = slight diarrhea, soft and loose feces, 2 = moderate diarrhea, semi-liquid feces, 3 = severe diarrhea, liquid and unformed feces^16^. Grouping criteria were performed as following: recording fecal consistency score of every defecation of each piglet, then summing and ranking total scores of each piglet, at last, combining with the clinical signs, the top five piglets with the highest and lowest fecal scores were designated as susceptibility (IS) and resistance (IR) groups, respectively.

The ileum tissues of fifteen piglets from IR, IS and IC groups were collected and flushed cleanly with PBS buffer (pH 7.4), and then quickly frozen in liquid nitrogen and stored at −80 °C until RNA extraction. Concurrently, the body weight, heart, liver, spleen, lung and kidney of piglets in IR, IS and IC groups were recorded. Blood samples of each piglets were collected from precaval vein every day, and stored at −80 °C.

### Total RNA isolation, library construction and lncRNA sequencing

Total RNA samples were isolated from ileum tissues using TRIzol™ reagent (Invitrogen, USA) and quantified by Nanodrop equipment. Purity and integrity of RNA extracts were assessed using the NanoPhotometer® spectrophotometer (IMPLEN, CA, USA) and RNA Nano6000 Assay Kit of the Bionalyzer 2100 system (Agilent Technologies, CA, USA), which were then used for library preparation.

Approximately 3 μg rRNA-depleted RNA (Ribo-Zero RNA) was acquired from total RNA by Epicentre Ribo-zero™ rRNA Removal Kit (Epicentre, USA) and cleaned up by ethanol precipitation to prepare sequencing library. Subsequently, strand-specific RNA sequencing libraries were generated from Ribo-Zero RNA by NEBNext® Ultra™ Directional RNA Library Prep Kit for Illumina® (NEB, Ipswich, MA, UK) to capture all transcripts with and without poly A. The library fragments of preferentially 150–200 bp in length were purified with AMPure XP system (Beckman Coulter, Beverly, USA). At last, library qualities were assessed on the Agilent Bioanalyzer 2100 system.

After clustering using the TruSeq PE Cluster Kit v3-cBot-HS (Illumina^®^), RNA libraries were sequencing on Illumina Hiseq4000 platform (Illumina, San Diego, CA, USA) to generated 150 bp paired-end (PE150) reads at the Novogene Bioinformatics Institute (Beijing, China).

### Identification of different expressed lncRNA and mRNA

After quality control, the paired-end clean reads were mapped to the pig reference genome sequence (*Sus scrofa* 10.2) by TopHat2^17^ and were assembled by Scripture^18^ and Cufflinks^19^ in a reference-based approach.

The coding potentials of transcripts were predicted by four tools named Coding-Non-Coding-Index (CNCI)^20^, Coding Potential Calculator (CPC)^21^, Pfam-scan v1.3 (E-value < 0.001)^22^, and phylogenetic codon substitution frequency (phyloCSF) v20121028^23^ to distinguish mRNA from lncRNA. Transcripts, which were predicted by any one of these four tools, were filtered out, those without coding potential were defined as candidate lncRNA.

The FPKMs (fragments per kilo-base of exon per million fragments mapped) of lncRNA and mRNA were calculated by Cuffdiff^19^. Gene FPKMs were computed by summing FPKMs of transcripts in each group. Differential expression levels were determined using a model based on the negative binomial distribution model. Transcripts with a corrected *P*-value < 0.05 were assigned as significantly differentially expressed.

### Target gene prediction

*Cis* and *trans* analyses were used to predict the target genes of differentially expressed lncRNAs. The target genes of lncRNA in *cis* role were predicted by lncRNAs regulation on expression of their neighboring protein-coding genes, which were close to 10 K upstream and downstream regions of lncRNA^24^. The target genes of lncRNA in *trans* role were identified by expression levels of lncRNA and mRNA, the expressed correlation between lncRNAs and coding genes was calculated based on Pearson’s correlation coefficient, the Pearson’s correlation coefficient (|*r*| > 0.95) were selected.

In structure, lncRNAs can form special secondary structures to regulate expression of mRNAs. Thus, the secondary structures of lncRNAs were predicted based on the free energy using RNAFold web server online software (http://rna.tbi.univie.ac.at/cgi-bin/RNAWebSuite/RNAfold.cgi).

### Function enrichment prediction

Analyses of Gene Ontology (GO) enrichment and Kyoto Encyclopedia of Genes and Genomes (KEGG) signaling pathway (https://www.kegg.jp/kegg/kegg1.html) were applied to investigate the potential roles of differentially expressed lncRNA target genes and mRNAs. The *P* value < 0.05 were considered as significantly enriched.

### Real-time quantitative PCR (qPCR) validation

Total RNA samples of 15 ileum tissues used for RNA-seq were processed to synthesize cDNA using reverse transcriptase Kit (TaKaRa, Dalian, China). A total of 3 lncRNAs (LNC_001066, LNC_001186 and ENSSSCT00000032859) and 5 mRNAs (*TNFRSF11A*, *TLR8*, *IRAK3*, *LCP2* and *CYP1A1*) were used to perform used to qPCR detection for validating the accuracy of RNA sequencing.

In addition, blood samples of 15 piglets from IR, IS and IC groups were collected at the 1, 2, 3, 4 and 5 days after infection, respectively. After lysing red blood cells from blood, total RNA samples were isolated from leukocytes using TRIzol reagent, and then used to reverse transcribe and synthesize cDNA using reverse transcriptase Kit (TaKaRa, Dalian, China). Finally, four cytokine genes (interleukin *IL-1β*, interferon *IFN-α*, tumor necrosis factor *TNF-α*, and nuclear factor *NF-κB*) were selected to quantify relative-expression levels to explore changes of cytokines.

The specific amplification primers of these genes, cytokines and housekeeping *GAPDH* gene were designed using NCBI website BLAST online software (Supplementary Table S1), the qPCR detection was qualified using 2^−∆∆Ct^ value methods^25^. The qPCR reaction was performed in 20 µL system involved 9.5 µL 2 × SYBR Green Realtime PCR Master Mix (TaKaRa, Dalian, China), 1 µL forward and reverse primers, 1 µL cDNA and 7.5 µL RNase free ddH_2_O using LightCycler 480II Real-Time PCR System. The cycling conditions included an initial activation denaturation (95 °C for 3 min), and followed by 30 cycles (95 °C for 15 s (denaturation), 60° ± 1 °C for 15 s (annealing), 72 °C for 20 s (extension)). Each biological replicate was comprised of three technical replicates.

### Statistical analysis

The experimental data was displayed as the mean ± standard error of mean (SEM). One-way ANOVA was performed to calculate statistical significance followed by Duncan to independently compare each *C*. *perfringens* type C treatment group to the control group. All statistical analyses were conducted by using correlation test (Student’s *t*-test) using SPSS 18.0 (SPSS Inc., Chicago, IL, USA), and *P* < 0.05 or *P* < 0.01 represent significance level.

## Results

### Difference of body and organ index among IR, IS and IC groups

**Figure 1:**
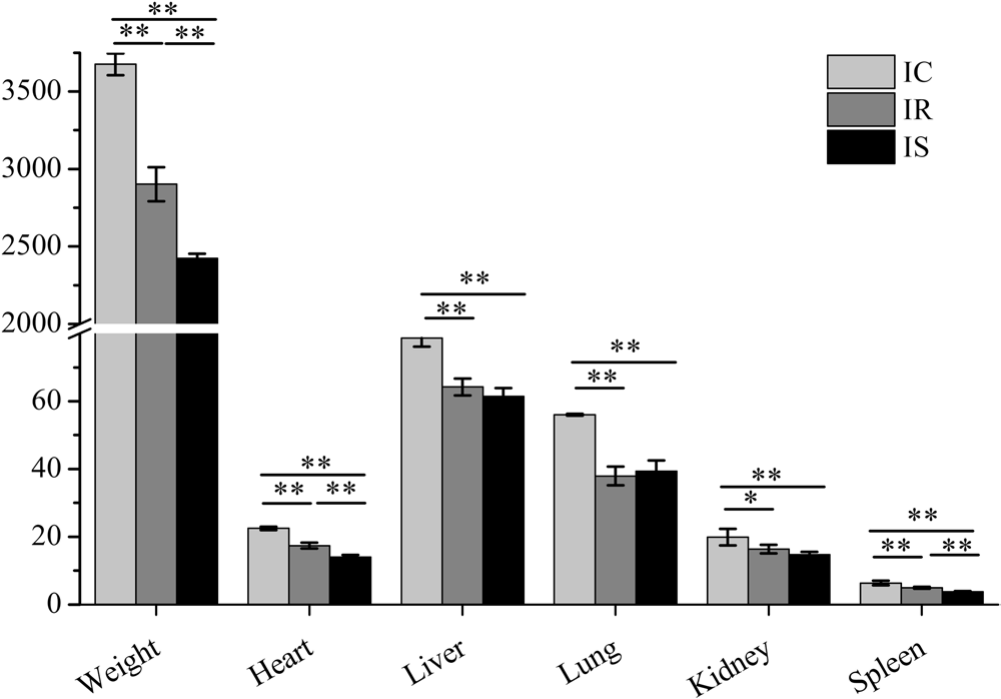
Comparison of body weight and organs index of piglets in IR, IS and IC groups after *C*. *perfringens* type C infection.

We had made the statistics analysis about the differences of body and organ weights of piglets among IR, IS and IC groups. As shown in Fig. 1, body and organ weights of infection group (IR and IS) were significantly lower than those of control group (*P* < 0.01), meanwhile, piglets in the IR group had significantly higher levels of body weight, heart, liver and spleen than piglets in the IS group (*P* < 0.01), however, the weights of liver, lung and kidney were not statistically significant difference between IR and IS groups (*P* > 0.05).

### Analyses of differentially expressed lncRNAs

**Figure 2:**
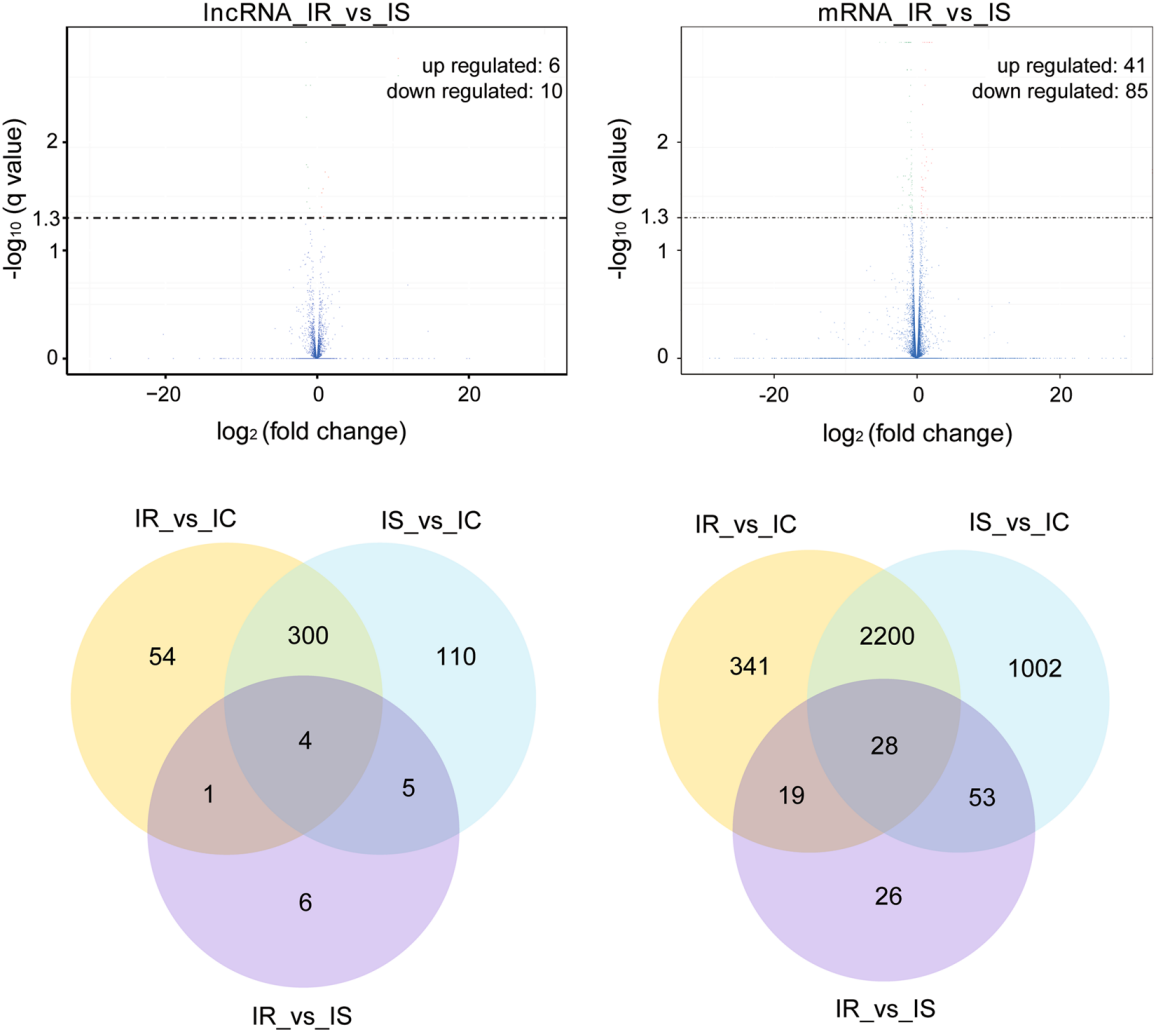
Analysis of differentially expressed lncRNAs and mRNA of piglets among the IR, IS and IC groups. (**A**) Venn diagram analysis of differently expressed lncRNA and mRNA; (**B**) Volcano plot analysis of differently expressed lncRNA and mRNA. Note: (1) Abscissa represents log2 (fold-change), and ordinate represents −log10 (*P* value); (2) Red dots denote the significant differentially expressed up-regulation and down-regulation transcripts, respectively; (3) Blue dots denote no differentially expressed transcripts.

To identify *C*. *perfringens*-responsive lncRNAs, the normalized expression of lncRNAs was compared between the IR and IS treatment groups. After *C*. *perfringens* type C infection, a total of 359 lncRNAs and 2588 mRNAs were significantly expressed in the IR vs IC group (Supplementary Table S2, Fig. 2A), as well as 419 lncRNAs and 3283 mRNAs in the IS vs IC group (Supplementary Table S3, Fig. 2A).

We conducted a Venn diagram for lncRNA and mRNA to understand the differential expression degree. As shown in Fig. 2A, a total of 16 lncRNAs and 126 mRNAs were found to differently express in the IR vs IS group, in which, 6 lncRNAs and 41 mRNAs were up-regulated, 10 lncRNAs and 85 mRNAs were down-regulated (Fig. 2B,C). There were 4 lncRNAs and 28 mRNAs were common differentially expressed among the IR, IS and IC groups after *C*. *perfringens* type C infection. Compared with IC group, 10 lncRNAs and 100 mRNAs were significantly differentially expressed in the IR vs IS group, which were used as the main resource for next analyses, and the details of these 10 lncRNAs were summarized and presented in Table 1.

**Table 1:** Information of 10 differentially expressed lncRNAs between IR and IS groups.

LncRNA transcript ID	LncRNA Gene ID	IR FPKM	IS FPKM	log2 (fold change)	*P* value	q value	Gene Location	Length (bp)
LNC_000139	XLOC_006938	7.038	2.40239	1.55069	0.00185	0.020992	GL895087.1:2-1569	509
LNC_001415	XLOC_076543	0.396698	0.885284	−1.1581	0.0014	0.016986	chr6:3126229-3131829	4595
LNC_000231	XLOC_012453	0	4.95443	—	5.00E-05	0.001192	JH118940.1:147819–149861	281
LNC_001186	XLOC_064823	1.68395	4.27736	−1.34487	0.0013	0.01609	chr3:142902784-142907208	2505
LNC_001066	XLOC_058106	1.38004	0.862734	0.67772	0.0029	0.029343	chr2:120605886-120628118	13522
ENSSSCT00000018610	ENSSSCG00000017092	20.8123	36.2241	−0.79951	0.00015	0.002973	chr16:78282293-78290916	1582
ENSSSCT00000032859	ENSSSCG00000030767	7.59312	20.0681	−1.40214	5.00E-05	0.001192	chr7:24721025-24724165	1147
ALDBSSCT0000004597	ALDBSSCG0000002795	9.56995	6.10481	0.648565	0.00435	0.039673	chr15:125123424-125136267	2047
ALDBSSCT0000009442	ALDBSSCG0000005758	88.3719	182.547	−1.04661	0.00255	0.026609	chr6:82227999-82233084	233
ALDBSSCT0000007865	ALDBSSCG0000004760	12.7032	33.9334	−1.41752	0.00015	0.002973	chr4:9144984-9146322	363

### Prediction of lncRNA and mRNA functions

To better understand the functions of lncRNAs, we firstly predicted the potential target genes of 10 lncRNAs, and meanwhile screened the differentially expressed target genes in IR vs IS groups compared to IC group, the screened results were presented in the Supplementary Table S4. While, there were no target genes predicted by three lncRNAs, LNC_000139, LNC_001415 and ALDBSSCT0000004597, which may be the reason of incomplete information for the updating pig genomic annotation.

**Figure 3:**
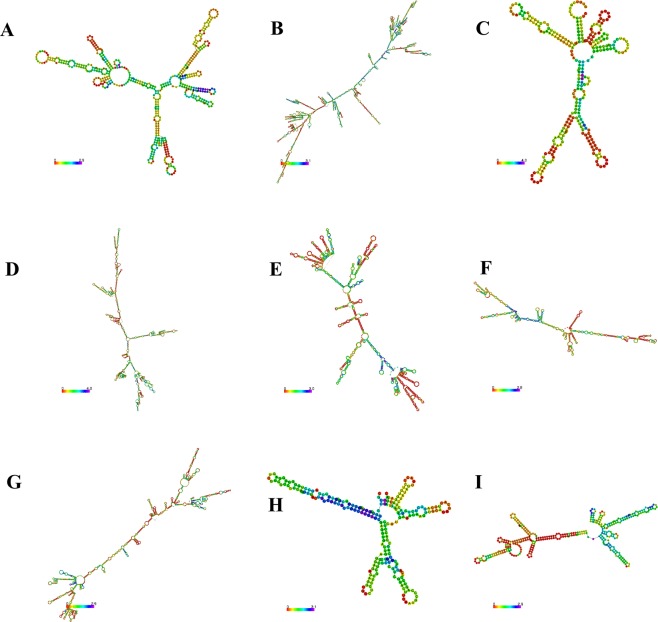
The secondary structures prediction of 9 screened differentially expressed lncRNAs. Note: A–O represents lncRNAs: LNC_000139, LNC_001415, LNC_000231, LNC_001186, ENSSSCT00000018610, ENSSSCT00000032859, ALDBSSCT0000004597, ALDBSSCT0000009442 and ALDBSSCT0000007865, LNC_001066 is not predicted for the length more than 10000 nt.

In general, lncRNA can act as signal molecules to regulate downstream gene transcription, as decoy molecules to play a role in blocking molecules, as guide molecules to combine with proteins, or as scaffold molecules to accurately control signal transduction and molecular dynamics in multiple biological processes. LncRNA exert regulatory function by three kinds of modes, i.e. local single chain structure, local secondary structural motifs, and target molecular interaction of particularly tertiary structural motifs^26,27^. Recently, it is difficult to acquire tertiary structure of lncRNA, secondary structure prediction of lncRNA target molecules are helpful to study their functional mechanisms in some degree, lncRNA with different secondary structures can exert different functions. Therefore, the structural properties of these 10 lncRNAs were predicted by statistical analyses, which may help to verify the subsequent function and to increase our understanding to their regulation modes. Finally, 9 differentially expressed lncRNAs were successfully predicted secondary structures (Fig. 3), except for LNC_001066 with excessive length. As demonstrated, the secondary structures of these lncRNAs mainly include stem loop, hairpin loop, multibranch loop *et al*., these loops are formed by with many unpaired bases, which can match with small molecules to regulate functions of RNAs and compounds by base pairing, thus each lncRNA may regulate multiple target genes.

**Figure 4:**
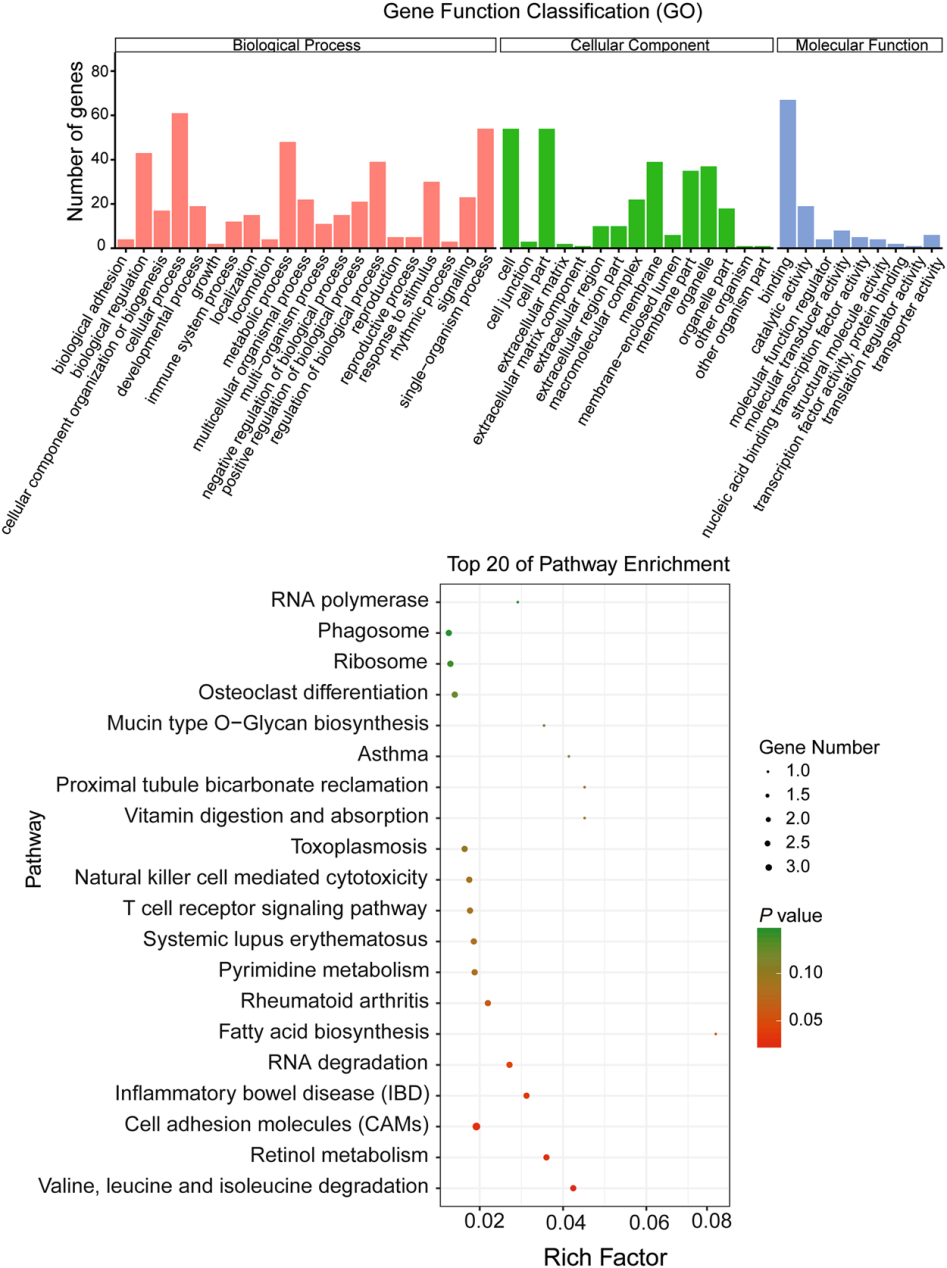
Functional enrichment analyses of lncRNA target genes identified in IR vs IS group. (**A**) GO function annotation of the screened lncRNAs target genes. The x-axis indicates the detail terms and the y-axis indicates gene numbers. (**B**) KEGG signaling pathways of the screened lncRNAs target genes. The x-axis indicates the gene ratio and the y-axis indicates the name of the KEGG pathway. The size of the dot indicates the number of target genes, and the color of the dot indicates different *P* value (Fisher’s Exact Test).

Furthermore, the GO and KEGG enrichment analyses were performed to predict the potential functions of lncRNAs. Except for 3 lncRNAs without predicted target genes, the remaining 7 lncRNAs were performed to predict target genes, which were presented in Table 2. These target genes were found to significantly enrich in 86 and 204 GO terms through *cis* and *trans* functions, respectively, in IR vs IS treatment group compared to IC group. In which, the main immune associated GO functions included MHC class I protein complex and transcription factor activity in cellular component, immune response, macrophage tolerance induction, antigen processing and presentation, and regulation of I-κB kinase/NF-κB signaling in biological process (Fig. 4A) (Supplementary Table S5), the top 20 enriched pathways of lncRNA target genes mainly included some immune and inflammatory related pathways, such as cell adhesion molecules, inflammatory bowel disease (IBD), T cell receptor signaling pathway, natural killer cell mediated cytotoxicity (Fig. 4B) (Supplementary Table S6).

**Table 2:** Target gene prediction of differentially expressed lncRNAs detected in the IRvsIS group.

Transcription ID	Target gene in *cis*	Target gene in *trans*
*LNC_000231*	*ZC3H7A*	***ABCA1***, *ALPK1*, *ANAPC13*, *BCL2L10*, *BTG4*, ***CD48***, *CLDN25*, *CRYBB3*, *DAZL*, *FOXR1*, *HMGB2*, *HSD17B10*, ***IL18RAP***, *KDM6A*, *KPNA7*, *LAMB3*, *LENG8*, *MAGEB3*, *MRC2*, *S100A16*, *S100A6*, ***SLA-DRB1***, *SLC39A4*, *SMARCA1*, *TCF4*, *TMEM187*, *TMEM244*, *FYB*, ***TNFRSF11A***, *TRIM77*, *UXT*, *WDR88*, *ZNF684*, *ZP4*, ***CD84***, *ENTPD1*, *IRAK3*, *MYCBP2*, *RASAL2*, ***TLR8***, *RPL39*, ***CYP1A1***, *PIP5KL1*, *TFCP2L1*
*LNC_001186*	***CYP1A1***, *PIP5KL1*, *TFCP2L1*	
*LNC_001066*		***LCP2***, ***CD84***, *ENTPD1*, *IRAK3*, *MYCBP2*, *RASAL2*, ***TLR8***
*ENSSSCT00000018610*	*TNIP1, MARCH6*	
*ENSSSCT00000032859*	*TMP-CH242-74M17*.*2*, ***SLA-1***, *TRIM26*, *CH242-196B23*.*2*	
*ALDBSSCT0000009442*	*SPOCD1*	
*ALDBSSCT0000007865*	*EFR3A*, *OC90*	

Note: The black represents the immune associated target genes of lncRNA, the underlined letter represents *C*. *perfringens* infectious diseases associated genes.

In addition, we further analyzed the enriched GO terms and KEGG pathways of 100 differentially expressed mRNAs. Results showed that a total of 19 significantly enriched GO terms (Corrected *P* Value < 0.05) and 549 GO terms were detected in IR vs IS treatment group (Supplementary Table S7). For example, the significantly enriched GO terms were mainly found in biological process of mucosal immune response, innate immune response in mucosa, immune response, defense response to gram-positive bacterium, and in cellular component of extracellular region. Meanwhile, 12 significantly enriched KEGG pathways main including toll-like receptor signaling pathway, antigen processing and presentation, glycosphingolipid biosynthesis, protein digestion and absorption, as well as some immune-related pathways of chemokine signaling pathway, NF-κB signaling pathway, cytokine-cytokine receptor interaction, and MAPK signaling pathway (*P* < 0.05) (Supplementary Table S8). Importantly, some immune-related genes were also found significantly differentially expressed and enriched in these bacterial infection associated pathways, such as *TLR8*, *LBP* and *SPP1* in toll-like receptor signaling pathway, *HSP70* and *CD8A* in antigen processing and presentation, *CXCL9*, *CXCL10*, *CCR5* and *CCL17* in chemokine signaling pathway.

**Figure 5:**
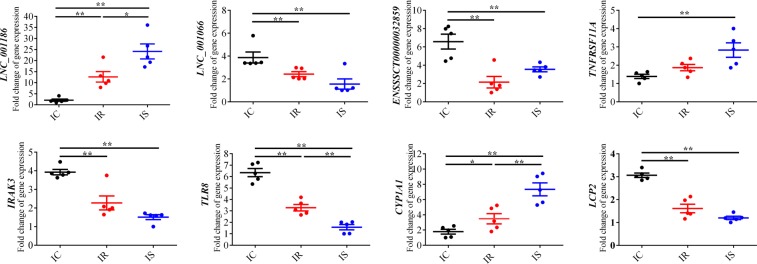
Expression level detection of 3 dysregulated lncRNAs and 5 dysregulated target mRNAs by qPCR method. Relative quantification of transcript expression was evaluated using the comparative cycle threshold (2^−∆∆Ct^) value method. The data were shown as mean ± SEM.

To validate the accuracy lncRNA sequencing data, a total of 3 lncRNAs and 5 mRNAs were selected to perform the qPCR detection. As shown in Fig. 5, the results of qPCR detection were perfectly matched to sequencing data, suggesting that the sequencing data was accurate.

### Expression of cytokine genes

**Figure 6:**
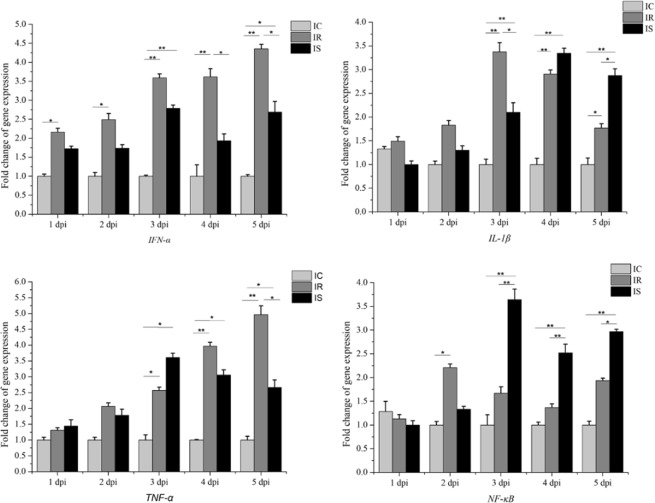
Expression levels of cytokines *IL-1β*, *IFN-α*, *TNF-α* and *NF-κB* in piglet blood after *C*. *perfringens* type C infection. The results are showed as mean ± SEM. Different asterisk above bars indicate significant differences (*P < 0.05, **P < 0.01).

The relative expressions of *IL-1β*, *IFN-α*, *TNF-α*, and *NF-κB* over the course of the infection were detected by qPCR method (Fig. 6). Compared to IC group, the expression levels of *IL-1β* and *NF-κB* were downregulated both in the IR and IS groups at the 1dpi, while *IFN-α* and *TNF-α* were upregulated in the IR and IS groups. in which, the expression of *IFN-α* was significant difference between the IR group and IC group (*P* < 0.05). At the 3dpi, expression levels of *TNF-α* and *NF-κB* in the IS group and *IL-1β* in the IR group were significantly increased in comparison with the IC group (*P* < 0.05). At the 5dpi, in the IR group, the expression levels of *IFN-α* and *TNF-α*were significantly upregulated than those in the IS group, while expressions of *IL-1β* and *NF-κB* in the IR group were significantly downregulated than those in the IS group. The results showed that *C*. *perfringens* type C infection significantly affect the expressions of these cytokine genes in piglets among IR, IS and IC groups, the high expressed cytokines may hint various degrees of inflammation response in piglets, which may be associated with the piglet immune resistant to *C*. *perfringens* type C infection.

## Discussion

*C*. *perfringens* has been recognized as one of the widespread potential bacterial pathogens, which can result in many infectious diseases, seriously influencing human and animal heaths. *C*. *perfringens* type C can cause piglet hemorrhagic diarrhea, even in human. Generally, the occurrences of these diseases are mainly caused by the close contact with *C*. *perfringens* type C-infected piglets or -polluted environments. The pathogenic ability of *C*. *perfringens* type C is various in infecting different piglet individuals and causing inflammatory diseases, in turn, piglets also have multifarious immune abilities to resist *C*. *perfringens* type C infection, these differences largely depend on host-pathogen interactions and host immune tolerance to bacteria^28^. Recently, lncRNA are considered as an important regulatory factor in modulating host inflammatory and immune responses against bacterial and viral infection^27,29,30^. Therefore, identifying *C*. *perfringens* type C resistant-associated lncRNAs and mRNAs and these potential functions in defensing bacterial infection were essential to further explore regulatory mechanism of host and prevention infectious diseases caused by *C*. *perfringens* type C.

*C*. *perfringens* type C infection cause some inflammatory and immune diseases, while the intensity and persistence of these diseases were associated with the resistance differences of piglet individuals to pathogen infection. Indeed, *C*. *perfringens* type C infection have affected the growing development of inoculated piglets, the inoculated piglets exhibited the growth delay and deficiency, the weights of body, heart, liver and spleen of piglets in the IR group were higher than those in the IS group (*P* < 0.01), suggesting that potential resistant piglets showed fewer adverse effects on growth and development than sensitive piglets. In addition, *C*. *perfringens* type C infection had changed the expression levels of several inflammatory cytokine genes. The dysregulated expressions of cytokines play a positive role in contributing to host immune and inflammatory responses in the intestine damage and host defense the invasion of various microorganisms, which may be associated with many autoimmunity diseases^31–35^. The *IFN-α*, *TNF-α*^31^, *IL-1β*^32^ and *NF-κB*^33^ are crucial mediators of inflammation, researches had reported that *TNF-α* and *IL-1* could induce biological activities of *IL-8* in various types of inflammation^34^. In our study, the overexpressed *IFN-α*, *TNF-α* and downregulated of *IL-1β* and *NF-κB* in the IR group were significantly different with these in the IS group after *C*. *perfringens* type C infection. The results showed that the lower inflammatory responses were activated in the resistant piglets, in other words, resistant piglets may have the better ability in reducing inflammatory responses induced by *C*. *perfringens* type C infection and may be more beneficial for improving their prognosis.

A total of 10 lncRNAs and 100 mRNAs were found significantly differentially expressed between IR vs IS group. We found that after *C*. *perfringens* type C infection, the differentially expressed lncRNAs LNC_001066, LNC_000231, LNC_001186 and ENSSSCT00000032859 were found to regulate some immune-related target genes interleukin 1 receptor associated kinase 3 (*IRAK3*), *TLR8*, *LCP2*, *TNFRSF11A*, *CYP1A1* and *SLA-1*, meanwhile, these target genes were also significantly differentially expressed in the IR vs IS group, the differentially expressed lncRNA target genes were mainly enriched in some key signal transduction and immune-related signaling pathways, such as toll-like receptor signaling pathway, NF-κB signaling pathway and cytokine-cytokine receptor interaction, which were considered to be associated with bacterial infection, especially *C*. *perfringens*^36,37^. These results may hint the potential effects of lncRNAs and target genes on signal transduction and cytokine. To further explore the potential functions of lncRNAs, we predicted the possible regulatory relationships by constructing a network diagram, which based on the following criteria: firstly, the immune related target genes of differentially expressed lncRNAs in *cis* and *trans* roles were predicted through target gene prediction, then we search the downstream inflammation related cytokine genes of immune target genes through KEGG signaling pathway database and some related articles. Based on the methods, a potential relationship among the 7 lncRNAs, target genes and inflammation related cytokines was evaluated and presented in the Supplementary Figure. As shown, the differentially expressed lncRNAs had potential regulatory relationship with their target genes. Furthermore, these target genes directly or indirectly affected the expressions of inflammation related cytokine genes *IL-1β*, *IFN-α*, *TNF-α*, *NF-κB*, *CCL5*, and so on, for example, the upregulated LNC_001066 could improve the expression of target genes *TLR8*, *IRAK3* and *LCP2*, overexpression of *TLR8*, *IRAK3* and *LCP2* genes directly or indirectly affected the expressions of *TRAF6*, *NF-κB* and *MAPK*, respectively, which may trigger the secretions of cytokines *IFN-α*, *IL-8* and *TNF-α* through NF*-*κB and MAPK signaling pathways. LNC_000231 could directly upregulated the expression of *TNFRSF11A*, *TRAF3* gene could be activated by the upregulated *TNFRSF11A*, and further affect the expression level of *NF-κB*, all of them participated in regulating host inflammatory and immune responses to *C*. *perfringens* type C infection. This analysis was performed to reveal the potential relationship between the differentially expressed lncRNAs, target genes and cytokine changes, which might provide some new perspectives for understanding the transcriptional regulation of piglet immune response to bacterial infection.

After *C*. *perfringens* type C infection, the expressions of LNC_001066 in the IR group were significantly upregulated compared with IS group, while the expressions of LNC_000231 was only found expressed in the IS group, the expressions of *IRAK3* and *TLR8* in the IR group were significantly higher than those in the IS group. To further explore the regulatory roles of lncRNAs, we predicted the potential relationship between lncRNA, target genes and cytokines. As shown in the Table 2, LNC_001066 and LNC_000231 co-regulated the expressions of immune-related target genes, *IRAK3* and *TLR8*, to participate in the progress of host immune response in defensing bacterial infection through apoptosis and toll-like receptor signaling pathway, respectively. *TLR8* is one of toll-like receptors (TLRs) family, which is the first line of host defense against invading pathogen invasion. *TLR8* can recognize distinct pathogen-associated molecular patterns in intracellular and play a critical role in host innate immune responses after bacterial infection^38^. *TLR8* can activate a series of cascade reaction, including inducing the expressions of *IRAK* and *TRAF6*^39,40^, and improve the productions of *IFN-α*, *NF-κB* through NF-κB and MAPK signaling pathways^41^. As the core immune regulator, the activations of NF-κB and MAPK pathways trigger the expressions of proinflammatory cytokines and chemokines, including the secretion of *TNF-α*, *IFN-α*, *IL-1β* and *IL-8*^38,42^. The suppressed TLRs could reduce systemic inflammatory response caused by bacterial infection. It was predicted that *TLR8* had potential relationship with the expressions of cytokines *IFN-α* and *IL-1β* through mediating expressions of *TRAF6*. *C*. *perfringens* infection suppressed the expression of *TLR8* in inoculated piglets, which was relatively higher expressed in IR group than that in IS group, interestingly, *IFN-α* was correspondingly higher expressed in IR group than in IS group, as well as expression of *IL-1β* at 1–3 dpi.

Beside this, LNC_000231 could target expression of *TNFRSF11A* gene, as known as receptor activator of NF-κB (*RANK*). *TNFRSF11A* can affect intracellular signal transduction process through several *TRAFs* and may lead to the activation of various signaling pathways, including the NF-κB, MAPK and PI3K/AKT cascades^43^. The overexpression of *TNFRSF11A* can interacted with TNF receptor-associated factors (*TRAF*) family members, such as *TRAF3* and *TRAF6* to activate the NF-κB-mediated apoptosis^44^. In our study, the expressions of *TNFRSF11A* were significantly upregulated after piglets inoculated with *C*. *perfringens* type C, and the expression in the IS group was higher than that in the IR group, suggesting that high expression of *TNFRSF11A* might trigger apoptosis process of intestinal cell^45^ and had an adverse effect in host defense the invasion of *C*. *perfringens* type C.

Lymphocyte cytosolic protein 2 (*LCP2*) is one of the immune genes targeted by LNC_001066 and involve in NK cell-mediated cytotoxicity pathway. *LCP2* plays important roles in promoting normal T-cell development and activation through MAPK signaling pathway^46^. Study found that the up-regulated *LCP2* could promote angiogenesis during precancerous lesion formation and activated the production of excessive amounts of proinflammatory cytokines, such as *IFN-γ* and *TNF-α*^47,48^, the loss of *LCP2* revealed a variable degree of abnormal intestinal vasculature in human and exhibited the hemorrhagic symptoms of subcutaneous and intraperitoneal tissues in mice^49^.The expression of *LCP2* in the IS group was lower than in the IR group, correspondingly, the expressions of *IFN-α* and *TNF-α* were also lower in the IS group, this results might cause some adverse symptom and resulted in the occurrence of damage lesions in *C*. *perfringens* type C infected piglets, including cell cycle regulation disorders and decreased local immunity^50^.

ENSSSCT00000032859 targeted the expression of *SLA-1* by antigen processing and presentation pathway, which was overexpressed in the IR group, and was not detected in the IS group. Study had reported that *SLA-1* could bind with toxic polypeptide and induced a series of immune responses by recognizing CD8^+^ cell, *E*. *coli* F18-resistant piglets exhibited high expression levels of *SLA-1*, which was related to several immune functions and could help to defense *E*. *coli* F18-related porcine gastrointestinal tract diseases^51^. The results also suggested that the high expression of *SLA-1* in resistant piglets may have the stronger capacity to defend *C*. *perfringens* type C infection.

The cytochrome P450 family 1 subfamily A member 1 (*CYP1A1*), directly targeted by LNC_001186, have some special relevance to metabolic activation and detoxication^52^, *CYP1A1* may stimulate the productions of inflammatory cytokines in immune and inflammatory responses, including *TNF-α*, *IL-1β*, *IL-6*, *CXCL5* and so on^53^. The activated *CYP1A1* is associated with tissue toxicity and carcinogenesis, such as gastrointestinal tract^54^. Study found that intestine *CYP1A1* contributed to a metabolic “shield” protecting host from ingesting carcinogens by interplaying with *TLRs* ligands^55^. Therefore, the higher expressed *CYP1A1* in the IS group might have some association with the expression of *IL-1β*, the higher expressed *IL-1β* reflected that the sensitive piglets might suffer more intense immune responses during *C*. *perfringens* type C infection.

In addition, *TNIP1* (*TNF*-*α* induced protein 3 (*TNFAIP3* or A20) interacting protein 1) could be targeted by ENSSSCT00000018610. Overexpression of *TNIP1* gene interact with *TNFAIP3* to restrict *NF-κB* activation, then further reduced autoimmunity response by TNF-independent signals^56^ and prevented intestine immune and inflammatory diseases^57–59^. Study reported that *TNFAIP3* repressed cell apoptosis by *TNF* and *IL-1β*, and affected proinflammatory gene expressions by directly acting on *TRAF6* signaling molecule^60^. *EFR3A* and *SPOCD1* gene were regulated by ALDBSSCT0000007865 and ALDBSSCT0000009442, respectively. *EFR3A* plays a role in controlling G protein-coupled receptor (GPCR) activity by affecting receptor phosphorylation, the mutation of this gene is associated with the early step of colorectal tumorigenesis^61^, changed expression of *EFR3A* contributes to host immune response against endocytosis of infectious bursal disease virus (IBDV) for preventing and decreasing infections^62^. *SPOCD1* protein product belongs to TFIIS family transcription factors, *SPOCD1* plays important functions in mediating inhibition of cancer cell proliferation^63^ and inducting cell apoptosis in breast, lung, gastric and pancreatic cancer cell lines through activation of p53^64^ and TGF-β signaling pathways^65,66^, the biological process involved by these genes ultimately plays a crucial role in the regulation of host inflammatory response.

The above results strongly implied the relationship among expression of lncRNA, target genes and cytokine changes, suggesting dysregulated lncRNAs affected inflammatory and proinflammatory cytokine expression by triggering the functional immune-related genes, meanwhile, expression differences of these molecules ultimately reflected in the resistance and susceptibility of host during pathogenic bacteria invasion. The piglets with higher resistance may have more abilities to reduce and counteract the damage caused by bacterial infection, though the definite information of how these lncRNAs regulate host immune response by affecting inflammatory cytokine changes need to be further validated.

## Conclusions

In this study, we have comprehensive compared the lncRNA expression patterns in intestinal inflammation response of piglets with different immune resistance in defensing *C*. *perfringens* type C infection. These significantly expressed lncRNA can trigger the target genes to influence cytokine expression, and further contribute to the different abilities of piglet resistance to bacterial infection or induce the injure of the inflammation diseases. This study will provide the pivotal resources for further exploring the resistance and susceptibility of hosts to *C*. *perfringens* infection.

## Supplementary information

supplementary information

Supplementary Table S1

Supplementary Table S2

Supplementary Table S4

Supplementary Table S4

Supplementary Table S5

Supplementary Table S6

Supplementary Table S7

Supplementary Table S8

## References

[1] Huang XY (2016). Effect of Genetic Diversity in Swine Leukocyte Antigen-DRAGene on Piglet Diarrhea. Genes..

[2] Songer JG, Pfeffer M, Truyen U, Gaastra W (2010). Clostridia as agents of zoonotic disease. Vet microbiol..

[3] Songer JG, Uzal FA (2005). Clostridial enteric infections in pigs. J Vet Diagn Invest..

[4] Chan G (2012). The epidemiology of Clostridium perfringens type A on Ontario swine farms, with special reference to cpb2-positive isolates. BMC Vet Res..

[5] Matsushita O, Okabe A (2001). Clostridial hydrolytic enzymes degrading extracellular components. Toxicon..

[6] Scharff RL (2012). Economic burden from health losses due to foodborne illness in the United States. J Food Prot..

[7] Popescu, F. *et al*. Susceptibility of primary human endothelial cells to C. perfringens beta-toxin suggesting similar pathogenesis in human and porcine necrotizing enteritis. *Vet microbiol*. **153**, 173–177, 10.1016/j.vetmic.2011.02.017, Epub 2011 Feb 23 (2011). 10.1016/j.vetmic.2011.02.017 21411248

[8] Zanella R (2011). Identification of loci associated with tolerance to Johne’s disease in Holstein cattle. Anim genet..

[9] Paralkar, V. R. *et al*. Lineage and species-specific long noncoding RNAs during erythro-megakaryocytic development. *Blood*. **123**, 1927–1937, 10.1182/blood-2013-12-544494, Epub 2014 Feb 4 (2014). 10.1182/blood-2013-12-544494 PMC3962165 24497530

[10] Zhao W (2015). Systematic identification and characterization of long intergenic non-coding RNAs in fetal porcine skeletal muscle development. Sci Rep..

[11] Kretz, M. *et al*. Suppression of progenitor differentiation requires the long noncoding RNA ANCR. *Genes Dev*. **26**, 338–343, 10.1101/gad.182121.111, Epub2012 Feb 2 (2012). 10.1101/gad.182121.111 PMC3289881 22302877

[12] Zhou, Y. *et al*. Integrative Analysis Reveals Enhanced Regulatory Effects of Human Long Intergenic Non-Coding RNAs in Lung Adenocarcinoma. *J Genet Genomics*. **42**, 423–436, 10.1016/j.jgg.2015.07.001, Epub 2015 Jul 10 (2015). 10.1016/j.jgg.2015.07.001 26336799

[13] Cui, W. *et al*. Discovery and characterization of long intergenic non-coding RNAs (lincRNA) module biomarkers in prostate cancer: an integrative analysis of RNA-Seq data. *BMC genomics*. **16**, 7, S3, 10.1186/1471-2164-16-S7-S3, Epub 2015 Jun 11 (2015). 10.1186/1471-2164-16-S7-S3 PMC4474418 26100580

[14] Kim, D. K. *et al*. Transcriptional profiles of host-pathogen responses to necrotic enteritis and differential regulation of immune genes in two inbreed chicken lines showing disparate disease susceptibility. *Plos one*. **9**, e114960, 10.1371/journal.pone.0114960, eCollection2014 (2014). 10.1371/journal.pone.0114960 PMC4263703 25504150

[15] Sarson AJ (2009). Gene expression profiling within the spleen of Clostridium perfringens-challenged broilers fed antibiotic-medicated and non-medicated diets. BMC genomics..

[16] Yang QL, Kong JJ, Wang DW, Zhao SG, Gun SB (2013). Swine Leukocyte Antigen-DQA Gene Variation and Its Association with Piglet Diarrhea in Large White, Landrace and Duroc. Asian-Australas J Anim Sci..

[17] Kim D (2014). TopHat2: accurate alignment of transcriptomes in the presence of insertions, deletions and gene fusions. Genome Biol..

[18] Guttman, M. *et al*. Ab initio reconstruction of cell type-specific transcriptomes in mouse reveals the conserved multi-exonic structure of lincRNAs. *Nat Biotechnol*. **28**, 503–510, 10.1038/nbt.1633, Epub 2010 May 2 (2010). 10.1038/nbt.1633 PMC2868100 20436462

[19] Trapnell, C. *et al*. Transcript assembly and abundance estimation from RNA-Seq reveals thousands of new transcripts and switching among isoforms. *Nature Biotechnol*. **28**, 511–515, 10.1038/nbt.1621, Epub 2010 May 2 (2010). 10.1038/nbt.1621 PMC3146043 20436464

[20] Sun, L. *et al*. Utilizing sequence intrinsic composition to classify protein-coding and long non-coding transcripts. *Nucleic Acids Res*. **41**, e166, 10.1093/nar/gkt646, Epub 2013 Jul 27 (2013). 10.1093/nar/gkt646 PMC3783192 23892401

[21] Kong L (2007). CPC: assess the protein-coding potential of transcripts using sequence features and support vector machine. Nucleic Acids Res..

[22] Punta, M. *et al*. The Pfam protein families database. *Nucleic Acids Res*. **40**, D290–D301, 10.1093/nar/gkr1065, Epub 2011 Nov 29 (2012). 10.1093/nar/gkr1065 PMC3245129 22127870

[23] Lin MF, Jungrei I, Kellis M (2011). PhyloCSF_ a comparative genomics method to distinguish protein coding and non-coding regions. Bioinformatics..

[24] Bruegge, J.Z., Einspanier, R. & Sharbati, S. A Long Journey Ahead: Long Non-coding RNAs in Bacterial Infections. *Front Cell Infect Microbio*l. **7**, 95, 10.3389/fcimb.2017.00095 eCollection2017 (2017). 10.3389/fcimb.2017.00095 PMC5368183 28401065

[25] Livak KJ, Schmittgen TD (2001). Analysis of relative gene expression data using real-time quantitative PCR and the 2(−Delta Delta C(T)) Method. Methods..

[26] Wang KC, Chang HY (2011). Molecular mechanisms of long noncoding RNAs. Molecular Cell..

[27] Mercer TR, Dinger ME, Mattick JS (2009). Long non-coding RNAs: insights into functions. Nat Rev Genet..

[28] Laine, A. L., Burdon, J. J., Nemri, A. & Thrall, P. H. Host ecotype generates evolutionary and epidemiological divergence across a pathogen metapopulation. *Proc Biol Sci.* **281**, 10.1098/rspb.2014.0522 (2014). 10.1098/rspb.2014.0522 PMC4071543 24870042

[29] Carpenter, S. *et al*. A long noncoding RNA mediates both activation and repression of immune response genes. *Science*. **341**, 789–792, 10.1126/science.1240925, Epub 2013 Aug 1 (2013). 10.1126/science.1240925 PMC4376668 23907535

[30] Atianand MK (2016). A Long Noncoding RNA lincRNA-EPS Acts as a Transcriptional Brake to Restrain Inflammation. Cell..

[31] Leppkes, M., Roulis, M., Neurath, M. F., Kollias, G., Becker, C. Pleiotropic functions of TNF-α in the regulation of the intestinal epithelial response to inflammation. *Int Immuno*l. **26**, 509–515, 10.1093/intimm/dxu051, Epub 2014 May 12 (2014). 10.1093/intimm/dxu051 24821262

[32] Alnabhani Z (2015). Pseudomonas fluorescens alters the intestinal barrier function by modulating IL-1β expression through hematopoietic NOD2 signaling. Inflamm Bowel Dis..

[33] Zhang, G. L. *et al*. Association of the NFKBIA gene polymorphisms with susceptibility to autoimmune and inflammatory diseases: a meta-analysis. *Inflamm Res*. **60**, 11–18, 10.1007/s00011-010-0216-2, Epub 2010 May 21 (2011). 10.1007/s00011-010-0216-2 20495844

[34] Miyamoto K, Matsukawa A, Ohkawara S, Takagi K, Yoshinaga M (1997). IL8 is involved in homologous TNF-α, but not in IL1ß-induced neutrophil infiltration in rabbits. Inflamm Res..

[35] Baccala R, Hoebe K, Kono DH, Beutler B, Theofilopoulos AN (2007). TLR-dependent and TLR-independent pathways of type I interferon induction in systemic autoimmunity. Nat Med..

[36] Vallabhapurapu S, Karin M (2009). Regulation and Function of NF-κB Transcription Factors in the Immune System. Annu Rev Immunol..

[37] Truong AD, Hong YH, Lillehoj HS (2015). RNA-seq profiles of immune related genes in the spleen of necrotic enteritis-afflicted chicken lines. Asian-Australas J Anim Sci..

[38] Lu Y (2009). Expression profiles of genes in Toll-like receptor-mediated signaling of broilers infected with *Clostridium perfringens*. Clin Vaccine Immunol..

[39] Kawai, T. & Akira, S. The role of pattern-recognition receptors in innate immunity: update on Toll-like receptors. *Nat Immunol*. **11**, 373–384, 10.1038/ni.1863, Epub 2010 Apr 20 (2010). 10.1038/ni.1863 20404851

[40] Heil F (2004). Species-specific recognition of single-stranded RNA via toll-like receptor 7 and 8. Science..

[41] Sloane, J. A., Blitz, D., Margolin, Z. & Vartanian, T. A clear and present danger: endogenous ligands of Toll-like receptors. *Neuromolecular Med*. **12**, 149–163, 10.1007/s12017-009-8094-x, Epub 2009 Oct 14 (2010). 10.1007/s12017-009-8094-x PMC2908951 19830599

[42] Cherfils-Vicini, J. *et al*. Triggering of TLR7 and TLR8 expressed by human lung cancer cells induces cell survival and chemoresistance. *J Clin Invest*. **120**, 1285, 10.1172/JCI36551, Epub 2010 Mar8 (2010). 10.1172/JCI36551 PMC2846035 20237413

[43] Darnay BG, Besse A, Poblenz AT, Lamothe B, Jacoby JJ (2007). TRAFs in RANK signaling. Adv Exp Med Biol..

[44] Bharti AC, Takada Y, Shishodia S, Aggarwal BB (2004). Evidence that receptor activator of nuclear factor (NF)-kappaB ligand can suppress cell proliferation and induce apoptosis through activation of a NF-kappaB-independent and TRAF6-dependent mechanism. J Biol Chem..

[45] Knesebeck, A. V. D. *et al*. RANK (TNFRSF11A) Is Epigenetically Inactivated and Induces Apoptosis in Gliomas. *Neoplasia*. **14**, 526–534. PMID: 22787434 (2012). 10.1596/neo.12360 PMC3394195 22787434

[46] Cuadros M (2007). Identification of a Proliferation Signature Related to Survival in Nodal Peripheral T-Cell Lymphomas. J Clin Oncol..

[47] Siggs OM (2015). Quantitative reduction of the T cell receptor adapter protein SLP-76 unbalances immunity and immune regulation. J Immunol..

[48] Kanehisa M (2005). From genomics to chemical genomics: new developments in KEGG. Nucleic Acids Res..

[49] Abtahian F (2003). Regulation of Blood and Lymphatic Vascular Separation by Signaling Proteins SLP-76 and Syk. Science..

[50] Chen D, Yang K, Zhang G, Mei J, Xiang L (2011). Screen and analysis of key disease genes for precancerous lesions of oral buccal mucosa induced by DMBA in golden hamsters. Oncol Lett..

[51] Ye, L. *et al*. Investigation of the relationship between SLA-1 and SLA-3 gene expression and susceptibility to *Escherichia coli* F18 in post-weaning pigs. *Comp Immunol Microbiol Infect Di*s. **3**5, 23–30, 10.1016/j.cimid.2011.09.006, Epub 2011 Oct 22 (2012). 10.1016/j.cimid.2011.09.006 22019298

[52] Alexandrov, K., Rojas, M. & Satarug, S. The critical DNA damage by benzo(a)pyrene in lung tissues of smokers and approaches to preventing its formation. *Toxicol Let*t. **19**8, 63–68, 10.1016/j.toxlet.2010.04.009, Epub 2010 Apr 24 (2010). 10.1016/j.toxlet.2010.04.009 20399842

[53] Uno S (2006). Oral benzo pyrene in Cyp1 knockout mouse lines: CYP1A1 important in detoxication, CYP1B1 metabolism required for immune damage independent of total-body burden and clearance rate. Mol Pharmacol..

[54] Vogel, C. F. A. *et al*. Transgenic Overexpression of Aryl Hydrocarbon Receptor Repressor (AhRR) and AhR-Mediated Induction of CYP1A1, Cytokines, and Acute Toxicity. *Environ Health Perspec*t. **124**, 1071–1083, 10.1289/ehp.1510194, Epub 2016 Feb 5 (2016). 10.1289/ehp.1510194 PMC4937866 26862745

[55] Do, K. N., Fink, L. N., Jensen, T. E., Gautier, L. & Parlesak, A. TLR2 Controls Intestinal Carcinogen Detoxication by CYP1A1. *PloS on*e. 7, e32309, 10.1371/journal.pone.0032309, Epub 2012 Mar 19 (2012). 10.1371/journal.pone.0032309 PMC3307708 22442665

[56] Cheng LQ, Zhang DM, Chen B (2016). Tumor necrosis factor α-induced protein-3 protects zinc transporter 8 against proinflammatory cytokine-induced downregulation. Exp Ther Med..

[57] Murphy SF, Rhee L, Nero TM, Boone DL (2013). 736 Intestinal Epithelial-Cell (IEC) Specific Expression of Tumor Necrosis Factor Alpha-Induced Protein 3 (TNFAIP3) Reveals Vital Role for IECs in Mediating Intestinal Inflammation. Gastroenterology..

[58] Vereecke L (2011). PS2-048. A20/TNFAIP3 in intestinal homeostasis and inflammation. Cytokine..

[59] Vereecke, L., Beyaert, R. & Loo, G. V. The ubiquitin-editing enzyme A20 (TNFAIP3) is a central regulator of immunopathology. *Trends Immuno*l. **30**, 383–391, 10.1016/j.it.2009.05.007, Epub 2009 Jul 28 (2009). 10.1016/j.it.2009.05.007 19643665

[60] Boone DL (2004). The ubiquitin-modifying enzyme A20 is required for termination of Toll-like receptor responses. Nat Immunol..

[61] Zhou, D. *et al*. Exome Capture Sequencing of Adenoma Reveals Genetic Alterations in Multiple Cellular Pathways at the Early Stage of Colorectal Tumorigenesis. *Plos one*. **8**, e53310, 10.1371/journal.pone.0053310, Epub 2013 Jan 2 (2013). 10.1371/journal.pone.0053310 PMC3534699 23301059

[62] Hui, R. K. & Leung, F. C. Differential Expression Profile of Chicken Embryo Fibroblast DF-1 Cells Infected with Cell-Adapted Infectious Bursal Disease Virus. *PloS one*. **1**0, e0111771, 10.1371/journal.pone.0111771 eCollection2015 (2015). 10.1371/journal.pone.0111771 PMC4460012 26053856

[63] Liang, J., Zhang, H., Hu, J., Liu, Y. & Li, Z. SPOCD1 promotes cell proliferation and inhibits cell apoptosis in human osteosarcoma. *Mol Med Rep*. **17**, 3218–3225, 10.3892/mmr.2017.8263, Epub2017 Dec 12 (2018). 10.3892/mmr.2017.8263 29257309

[64] Hubbard K, Catalano J, Puri RK, Gnatt A (2008). Knockdown of TFIIS by RNA silencing inhibits cancer cell proliferation and induces apoptosis. BMC Cancer..

[65] Cha, Y., Kim, D. K., Hyun, J., Kim, S. J. & Park, K. S. TCEA3 binds to TGF-beta receptor I and induces Smad-independent, JNK-dependent apoptosis in ovarian cancer cells. *Cell Signa*l. **25**, 1245–1251, 10.1016/j.cellsig.2013.01.016, Epub 2013 Jan 26 (2013). 10.1016/j.cellsig.2013.01.016 23357533

[66] Zhu, M. *et al*. Exome Array Analysis Identifies Variants in SPOCD1 and BTN3A2 That Affect Risk for Gastric Cancer. *Gastroenterolog*y. **152**, 2011–2021, 10.1053/j.gastro.2017.02.017, Epub 2017 Feb 27 (2017). 10.1053/j.gastro.2017.02.017 28246015

