# Supplementary material for: Novel Insights reveal Anti-microbial Gene Regulation of Piglet Intestine Immune in response to Clostridium perfringens Infection
Source: Sci Rep. 2019 Feb 13;9:1963. doi: 10.1038/s41598-018-37898-5 (PMC6374412; doi:10.1038/s41598-018-37898-5)
Supplement: Supplementary file 1 — supplementary information [file 41598_2018_37898_MOESM1_ESM.pdf]

Novel Insights reveal Anti-microbial Gene Regulation of Piglet Intestine Immune in response to *Clostridium perfringens* Infection

Xiao Yu. Huang[1†], Wen Yang. Sun[1†], Zun Qiang. Yan[1], Hai Ren. Shi[1], Qiao Li. Yang[1], Peng Fei. Wang[1], Sheng Gui. Li[1], Li Xia. Liu[2], Sheng Guo. Zhao[1]and Shuang Bao. Gun[1,3*]

[1] College of Animal Science and Technology, Gansu Agricultural University, Lanzhou, China,
[2] College of Life Science and Engineering, Northwest University for Nationalities, Lanzhou, China,
[3] Gansu Research Center for Swine Production Engineering and Technology, Lanzhou, China

E-mail: huanghxy100@163.com (X.H.), sun_china@outlook.com (W.S.), yanzunqiang@163.com (Z.Y.), 1036523665@qq.com (H.S.), yangql0112@163.com (Q.Y.), wangpf815@163.com (P.W.), Lisg2014@163.com (S.L.), skllx@xbmu.edu.cn (L.L), zhaosg@gsau.edu.cn (S.Z.), gunsbao056@126.com (S.G.).

[*] Correspondence: Shuang Bao Gun, College of Animal Science and Technology, Gansu Agricultural University, No. 1 Yingmen village, Anning district, Lanzhou 730070, China. Tel: +86-931-7631804, Fax: +86-931-7632468, E-mail: gunsbao056@126.com.

† They were work equally to this study.

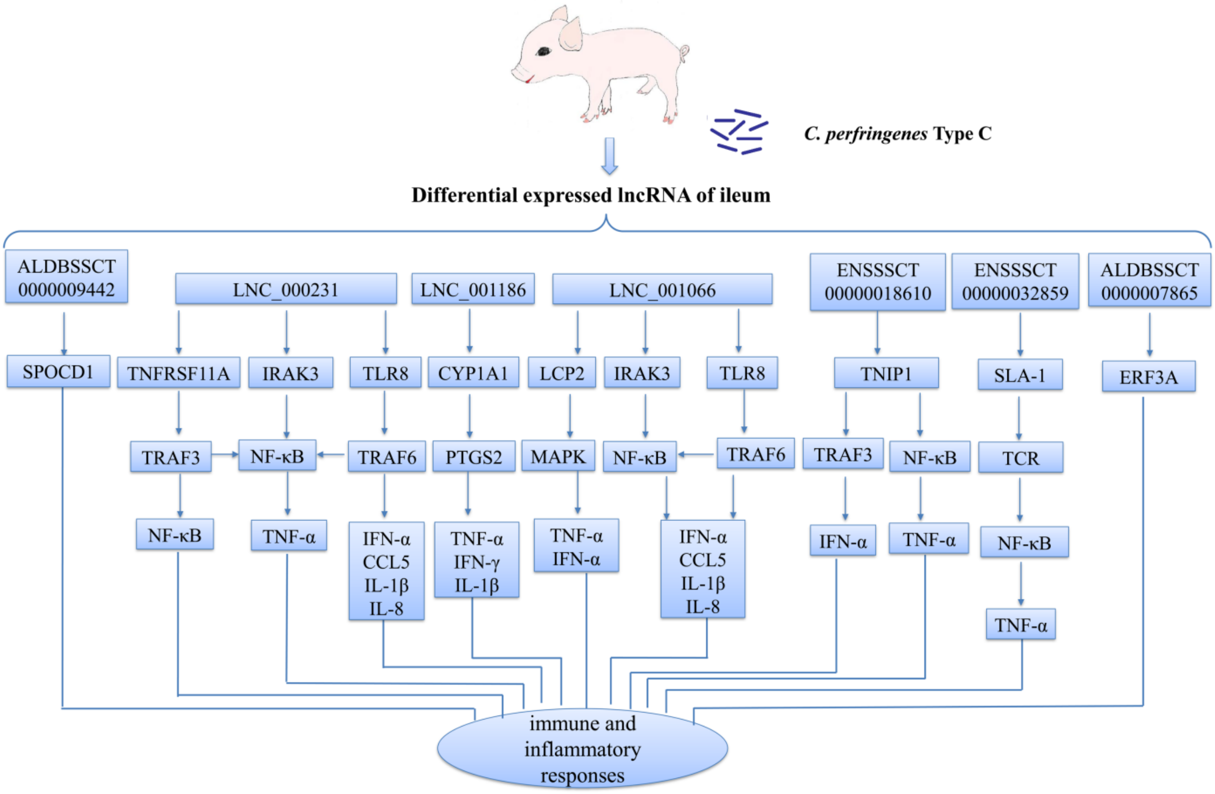

Supplementary Figure. Analyses of potential regulatory relationships among the differentially expressed lncRNAs, target genes and cytokines.

Supplementary Files:

Supplementary Table S1: Primer sequences of lncRNA, mRNA and cytokines used in qPCR detection.

Supplementary Table S2: Catalog of ileum differentially expressed lncRNAs and mRNAs identified in piglets from IR vs IC group.

Supplementary Table S3: Catalog of ileum differentially expressed lncRNAs and mRNAs identified in piglets from IS vs IC group.

Supplementary Table S4: Target gene prediction of ileum differentially expressed lncRNAs in piglets after *C. perfringens* Type C infection.

Supplementary Table S5: GO enrichment analysis of significantly expressed lncRNA target genes between IR and IS groups.

Supplementary Table S6: KEGG enrichment analysis of the significantly expressed lncRNA target genes between IR and IS groups.

Supplementary Table S7: GO enrichment analysis of significantly expressed mRNA between IR and IS groups.

Supplementary Table S8: KEGG enrichment analysis of the significantly expressed mRNA between IR and IS groups.

Supplementary Figure. Analyses of potential regulatory relationships among the differentially expressed lncRNAs, target genes and cytokines.
